# Nitric Acid for Hoarseness

**Published:** 1877-04

**Authors:** 


NITRIC ACID FOR HOARSENESS.—Dr. W. Handsell Griffiths says that a few drops of nitric acid in a glass of sweetened water, a couple of times daily, will be found an excellent remedy for the hoarseness of singers.—*Richmond and Louisville Medical Journal.*

